# The Effect of Aquatic Exercise Training on Heart Rate Variability in Patients with Coronary Artery Disease

**DOI:** 10.3390/jcdd9080251

**Published:** 2022-08-06

**Authors:** Borut Jug, Danijela Vasić, Marko Novaković, Viktor Avbelj, Lea Rupert, Juš Kšela

**Affiliations:** 1 Department of Vascular Diseases, University Medical Centre Ljubljana, 1000 Ljubljana, Slovenia; 2 Medical Faculty, University of Ljubljana, 1000 Ljubljana, Slovenia; 3 Centre for Cardiac Rehabilitation Šmarješke Toplice, 8220 Šmarješke Toplice, Slovenia; 4 Department of Communication Systems, Jozef Stefan Institute, 1000 Ljubljana, Slovenia; 5 Department of Anaesthesiology and Perioperative Intensive Therapy, University Medical Centre Ljubljana, 1000 Ljubljana, Slovenia; 6 Department of Cardiovascular Surgery, University Medical Centre Ljubljana, 1000 Ljubljana, Slovenia

**Keywords:** exercise training, aquatic (water-based) exercise, heart rate variability, coronary artery disease

## Abstract

*(1) Background:* Aquatic exercise training is a relatively understudied exercise modality in patients with CAD; with the present study, we sought to compare the impact of short-term 14-day water- and land-based exercise training on heart rate variability (HRV). *(2) Methods:* We randomized 90 patients after a recent CAD event (myocardial infarction and/or revascularization within 2 months prior to inclusion) to either (i) water-based or (ii) land-based exercise training (14 days, two 30 min sessions daily), or (iii) controls. Before and after the intervention period, all participants underwent 20 min 12-channel high-resolution ECG recordings with off-line HRV analysis, including conventional linear time- and frequency-domain analysis (using the Welch method for fast-Fourier transformation), and preselected non-linear analysis (Poincaré plot-derived parameters, sample entropy, and the short-term scaling exponent α1 obtained by detrended fluctuation analysis). *(3) Results*: Eighty-nine patients completed the study (mean age 60 ± 8 years; 20 % women). We did not detect significant differences in baseline- or age-adjusted end-of-study HRV parameters, but aquatic exercise training was associated with a significant increase in the linear LF/HF parameter (from 2.6 [1.2–4.0] to 3.0 [2.1–5.5], *p* = 0.046) and the non-linear α1 parameter (from 1.2 [1.1–1.4] to 1.3 [1.2–1.5], *p* = 0.043). *(4) Conclusions:* Our results have shown that a short-term 14-day aquatic exercise training program improves selected HRV parameters, suggesting this mode of exercise is safe and may be beneficial in patients with CAD.

## 1. Introduction

Exercise-based cardiac rehabilitation reduces cardiovascular morbidity and mortality in patients with coronary artery disease (CAD) [1,2,3]. Ample evidence suggests that exercise training improves markers of cardiovascular health such as risk factors, exercise capacity, vascular function and heart rate variability (HRV); however, the vast majority of exercise studies in patients with CAD employ land-based aerobic dynamic modalities, such as cycling or treadmill walking [3,4,5]. Conversely, aquatic exercise (such as swimming or water aerobics training)—while a popular exercise modality—remains rarely provided or even discouraged, mainly because of its perceived risks (i.e., unfavorable hemodynamic response to water immersion and temperature, possibly yielding ventricular dysfunction and/or dysrhythmias in cardiac patients) [6,7]. However, a growing body of evidence now suggests that aquatic exercises in patients with CAD may safely improve exercise capacity, vascular function and various biomarkers of cardiovascular health [8,9,10,11].

Heart rate variability (HRV) is a strong marker of cardiovascular health [12]. An indicator of cardiac autonomic modulation, HRV responds to physiological stimuli, such as exercise (therefore providing a measure for cardiovascular fitness), and is impaired by disease (therefore providing a tool for risk assessment and prognostic inference in various cardiovascular diseases) [13]. Unsurprisingly, exercise training improves HRV in healthy individuals, athletes and patients with cardiovascular diseases; however—again—most of the evidence on the effects of exercise on HRV modulation is derived from land-based exercise studies [14]. Water immersion and aquatic exercise elicit distinct and specific HRV responses in healthy individuals but have not been studied in CAD patients to date.

Therefore, we sought to appraise the effects of aquatic exercise training on HRV in patients undergoing short-term cardiac rehabilitation after a recent CAD event. We have previously demonstrated that aquatic exercise improves exercise capacity, vascular function and health-related quality of life [11]; in this sub-study, we hypothesized that short-term (14-day) aquatic exercise training would improve HRV parameters as compared to land-based training and controls (standard non-supervised exercise routine).

## 2. Materials and Methods

### 2.1. Study Design and Participants

This was a prospective, randomized, open-label parallel trial comparing water- and land-based exercise training with non-supervised training [11]. We included patients with CAD after a recent (<3 months) myocardial infarction and/or revascularization procedure (PCI or CABG) undergoing stationary cardiac rehabilitation. We excluded patients with uncontrolled/decompensated valve diseases, uncontrolled arterial hypertension, uncontrolled/high-risk dysrhythmias or the presence of a permanent pacemaker, contraindications to exercise, inability to perform exercise testing, mental impairment, severe anemia, severe obstructive/restrictive lung disease, recent thromboembolic events, hepatic dysfunction, and/or age over 80 years. Patients who met the inclusion criteria were invited to participate in the study. A written informed consent form was obtained for each participant. Power calculation suggested that 26 patients should be included in each group to detect a between-group difference of one standard deviation in any selected end-of-study measurement at a two-sided 0.05 significance level with the probability (power) of 80%. With a conservative estimate of a drop-out rate of 10–20%, we decided to include 30 patients in each group.

Patients were urn-randomized (using a sealed envelope method) to either water-based training, land-based training, or controls (i.e., not included in a supervised exercise training program, but advised to undertake low-to-moderate exercise routines at home). Patients received standard therapy for the treatment of ischemic heart disease. Principles of treatment over the period of research were not changed, but medicines were adjusted when it was necessary to ensure that blood pressure and heart rate were optimally controlled.

The study complied with the Declaration of Helsinki on ethics in medical research and was approved by the Republic of Slovenia Medical Research Ethics Committee (0120-655/2016-2) and was registered at the ClinicalTrials.gov number NCT02831829.

### 2.2. Intervention

The study intervention consisted of two short-term (14 days) exercise training programs with two 30 min daily sessions (24 sessions in total) performed at 60–80% of the peak heart rate achieved on the symptom-limited grade exercise test. At inclusion, all participants underwent standard symptom-limited pre-training cardiopulmonary bicycle testing using the cycle ergometer Schiller CS-200 (Schiller A.G., Baar, Switzerland) with an incremental ramp protocol to achieve the predicted maximal workload. This was considered completed if the respiratory exchange ratio was ≥1.1 using continuous ECG monitoring.

Water-based exercise training was performed in a heated swimming pool (32.8 °C), with patients exercising in an upright position at the level of the xiphoid process (depth 1.3 m); the daily program consisted of one water aerobics session (water walking, side-stepping, arm cycling, etc.) and one calisthenics session (triceps extensions, triceps dips, leg press, abduction and adduction, and wall push-ups). Land-based exercise training comprised one aerobic session (bicycle ergometer training) and one calisthenics workout. Patients in the control group were given lifestyle advice, including recommendations on the benefits of regular physical activity in CAD, but were asked to refrain from enrolling in a supervised exercise program for the duration of the intervention period (i.e., two weeks).

### 2.3. Cardiac Autonomic Modulation

Before and after the intervention period, all patients underwent a 20 min high-resolution ECG recording using a 12-channel digital recorder, Schiller CS-200 (Schiller A.G., Baar, Switzerland). The measurements were taken between 9 AM and 11 AM in the postprandial state, 10 min after supine rest in a quiet room kept at 22–24 °C with relative humidity between 40 and 70%. Patients were asked not to smoke or drink any caffeinated beverages 24 h prior to measurements.

First, off-line analysis of the R–R interval using R-peak detection was performed using the Schiller SEMA-200. Each detected beat was classified as normal, ventricular ectopic, supraventricular ectopic, or unknown. The location of the R-wave peak was determined with a resolution of 1 ms through interpolation. After automated editing, an experienced observer reviewed and corrected all tracings manually. Editing eliminated all abnormal beats, including the ones succeeding the ectopic beats in case of either ventricular or supraventricular ectopy without any interpolation attempted for eliminated intervals. Next, a moving-window average filter was applied to the edited data. For each set of five contiguous NN intervals, a local average was computed excluding the central interval. If the value of the central interval was 20% greater or smaller than the local average, it was considered to be an outlier and replaced by the local average. Only recordings with >95% pure sinus beats were included in the linear and non-linear HRV analysis.

For the HRV analysis, raw data were processed in Matlab (Math Work Inc., Natick, MA, USA) using the freely available software HRV Analysis Software (HRVAS) (available at http://sourceforge.net/projects/hrvas/, accessed on 5 July 2022) as described in detail elsewhere (Ramshur, 2010; Solca et al., 2018).

*Linear HRV analysis*—For time-domain HRV measures, the standard deviation of normal-to-normal RR intervals (SDNN), the standard deviation of the average normal-to-normal RR intervals (SDANN), the square root of the mean square differences of successive normal-to-normal RR intervals (RMSSD), and the mean standard deviation of normal-to-normal RR intervals for every 5 min (SDNNi) were calculated.

Frequency-domain linear HRV measures were obtained by using the Welch method for fast-Fourier transformation. Areas of spectral peak in ranges from 0.04 to 0.15 Hz and from 0.15 to 0.40 Hz were defined as low-frequency (LF) power indicating modulated sympathetic activity, and high-frequency (HF) power indicating vagal activity, respectively. The sympathovagal balance was calculated as the ratio of LF and HF powers (LF/HF ratio).

*Non-linear HRV analysis*—The standard deviation of the short- (SD1) and long-term (SD2) beat-to-beat RR interval variability measure (Poincaré plot), the quantification of self-similarity in an RR data set and distinguishing chaos from randomness by embedding a data set into a higher-dimensional state space, sample entropy (SE), measuring the complexity of the RR interval time series independently from the data length, and the short-term scaling exponent obtained by the detrended fluctuation analysis technique of the RR interval time series, which provides an estimation of fractal correlations in heart rate dynamics for short-term RR interval data sets (<11 beats, α1), were determined as non-linear measurements of HRV.

### 2.4. Statistical Analysis

Data are presented as mean (standard deviation) for normally distributed continuous variables and as median (interquartile range) if they lacked normal distribution. Between-group differences in the patients’ baseline characteristics were tested by ANOVA or the Kruskal−Wallis test, as appropriate. Differences in end-of-study HRV parameters were tested using ANCOVA with age and baseline measurements as covariates, and post hoc differences were tested by the Sidak test. Differences from the baseline within each group were also tested using a paired sample t-test for normally distributed differences, and the Wilcoxon test otherwise.

## 3. Results

**Figure 1:**
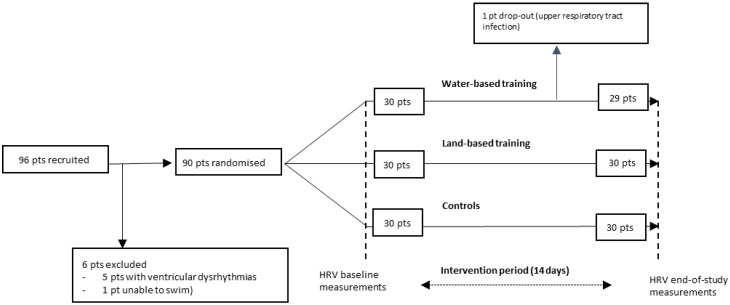
Patient flow chart.

We included 90 patients (96 patients were recruited, 6 were excluded—5 because of history of ventricular dysrhythmias, and 1 because of the inability to swim); 89 completed the study with 29 in the water-based group (1 dropped out because of upper respiratory tract infection in the water-based group), 30 in the land-based group and 30 in the control group (Figure 1). The mean age was 60 years, 25 % were women, and the median time from CAD event to inclusion was 37 days. Patients at inclusion received standard secondary prevention therapies, including beta blockers when not contraindicated (i.e., 85% patients).

There were no significant differences between the two intervention groups and the control group at baseline, except for age, with water-based participants being significantly older, thus suggesting randomization failure and necessitating age-adjustment in further analysis (Table 1).

### 3.1. Time-Domain HRV Parameters

Time-domain HRV parameters before and after the intervention period did not change significantly in either group, except for an increase in mean heart rate in the aquatic exercise training group (Table 2, complete set of measurements is available as Supplementary Table S1).

### 3.2. Frequency-Domain HRV Parameters

For frequency-domain parameters, we observed a statistically significant decrease in the LF/HF index in the water-based group (*p* = 0.036) (Table 3, complete set of measurements is available as Supplementary Table S2).

### 3.3. Non-Linear HRV Parameters

A significant increase in the non-linear HRV α2 parameter was observed in the land-based group (*p* = 0.003), and a significant increase in the non-linear HRV α1 parameter was observed in the water-based group (*p* = 0.043) (Table 4, complete set of measurements is available as Supplementary Table S3).

## 4. Discussion

Short-term 14-day aquatic exercise training in patients with CAD is associated with specific changes in HRV as compared to land-based training. In our study, aquatic exercise training was associated with a significant change in selected HRV parameters (i.e., increase in the linear frequency-domain parameter LF/HF and a decrease in the non-linear parameter α1). On the one hand, our results provide evidence on the distinct impact of aquatic exercise training on cardiac autonomic modulation; on the other hand, the specific pattern of HRV changes may suggest distinct cardiovascular benefits of water-based exercise modalities in patients with CAD.

Our study adds to the vast body of evidence of the effects of exercise training on HRV, addressing the specific role of aquatic exercise in CAD. The impact of exercise training on HRV parameters in healthy individuals, athletes and patients with cardiovascular diseases is well established [14,15,16], whereas the comparative effects of different exercise modalities in CAD are less well defined. So far, studies have shown that land-based exercise protocols can increase selected time-domain linear HRV parameters, suggesting beneficial effects on cardiovascular health in patients after myocardial infarction or revascularization [14,17,18]. Conversely, ours is the first trial to compare HRV changes in CAD after water-based training, land-based training, or unsupervised exercise routines.

In our study, short-term aquatic exercise was associated with detectable effects on HRV. Although the exact mechanisms are not entirely clear, possible explanations include methodological challenges, training duration and specific effects of water immersion and aquatic training intensity. In terms of methodological challenges, randomization failure resulted in older patients randomized to land-based exercise training, possibly retaining the effect of age on HRV even after statistical adjustment. In terms of duration, most studies detecting the impact of land-based training on HRV employed longer duration protocols (i.e., 6–8 weeks) [19], whereas our 14-day protocol might have been too short to elicit similar results in the land-based group. The relatively short duration may also provide a plausible explanation for lack of differences in end-of-study HRV parameters between study groups, as duration (along with intensity) seems to be a pivotal determinant of HRV response to exercise training [14]. In terms of water immersion and training intensity, aquatic exercise provides unique physiologic responses. On the one hand, water immersion is associated with specific workloads (due to the physical properties of water, such as buoyancy, resistance and/or viscosity) and hydrostatic pressure-induced cardiorespiratory responses [11,20]. On the other hand, peak-heart-rate-derived intensity may have underestimated the actual metabolic stress provided by aquatic exercise; while some evidence suggests that aquatic exercise may require intensity adjustments (‘aquatic correction’) [10], other evidence does not [8]. In our study, exercise training in deep (xiphoid process level) water, which is associated with higher buoyancy and lower resistance [20], likely resulted in comparable intensity to land-based training, but with a more pronounced hydrostatically induced hemodynamic response to immersion.

The specific pattern of HRV changes associated with aquatic exercise in CAD also merits addressing. HRV may be regarded either broadly as a marker of adaptability of cardiac autonomic modulation or specifically as a marker of sympathovagal balance [12]. The ‘adaptability’ interpretative framework regards an increase in HRV as a marker of cardiovascular health in and of itself, suggesting cardiac autonomic modulation may adequately respond to external stimuli, such as stress or exercise. Conversely, the ‘sympatovagal balance’ interpretative framework has been challenged, as the sympathetic and parasympathetic modulation of cardiac autonomic activity is more complex than what is captured in traditional linear HRV parameters, such as the LF/HF ratio [21]. In fact, in our study, 14 days of aquatic exercise training yielded an increase in both the linear HRV parameter LF/HF, which is traditionally regarded as a marker of sympathicovagal balance with a pronounced indication of sympathetic activity, and the non-linear α1, which more broadly captures parasympathetic predominance and is inversely associated with cardiovascular risk [12,22]. In this regard, current physiologic understanding suggests that the LF/HF ratio oversimplifies the complex sympathetic and parasympathetic effects on cardiac autonomic modulation [21,23], and thus cannot be regarded as a reliable and sound marker of sympathovagal equilibrium. Conversely, non-linear parameters, such as α1—derived from the mathematics of complex dynamics and fractal geometry—may provide a more precise estimate of the sympathovagal balance [24]. In line with this physiologic understanding, our findings suggest that short-term aquatic exercise training may improve sympathovagal balance (and consequently the complex dynamics of HRV), which is in line with the vast evidence of training-induced adaptations of cardiac autonomic function [13,14,19]. In addition, our study observations are limited to the ECG-derived appreciation of cardiac autonomic modulation, whereas physiologic responses to exercise encompass complex cardiac–peripheral vascular coupling. For instance, the photoplethysmographic appreciation of pulse wave recordings might have provided complementary information on the interaction between regulatory processes of cardiac and peripheral vascular functions, and presented a more complete appraisal of the effects of different exercise modalities on overall autonomic functions [25,26,27], but were not appraised as part of our study.

Our study has several limitations. Firstly, this was a single-center study in selected CAD patients and cannot be extrapolated to patients with other cardiovascular conditions, such as heart failure. Secondly, the between-group age differences at baseline indicate randomization failure; as this required statistical adjustments, our study should be regarded as a pilot hypothesis-generating study. Thirdly, the intervention effects of both intervention groups in comparison to controls may have been overestimated, because both intervention groups participated in the residential cardiac rehabilitation (comprehensively addressing other risk factors beyond exercise training) and the control group did not. Moreover, the control group—while not undergoing supervised exercise training—was made aware of the beneficial effects of regular exercise, which was not controlled for; lack of adequate measurement of unsupervised physical activity in the control group is a major limitation of our study. Lastly, exercise intensity prescription was derived from peak heart rate during symptom-limited bicycle testing. On the one hand, the reliability of such a mode for metabolic stress inference in cardiovascular patients on beta blockers (i.e., such as our study population) may be less reliable than ventilatory threshold appraisal [28]. However, the majority of studies—ours included—still favor the peak heart rate method because it remains readily applicable and replicated in clinical practice [29]. On the other hand, intensity prescription derived from a land-based exercise test may have underestimated the water-based training intensity. The specific type (xiphoid-level endurance *plus* calisthenics exercise training) and duration of our program (two weeks) allowed us to only explore immediate physiological responses, without focusing on any sustainable effects of regular training. Considering immersion and intensity, ours was a study of deep-water (xiphoid level) training without heart rate aquatic adjustment. For this reason, we advise caution when attempting to extrapolate our results to other forms of aquatic exercise or compare the intensity of water- vs. land-based training.

## 5. Conclusions

This is the largest study comparing water- and land-based exercise training (vs. unsupervised exercise routine controls) in patients after a recent CAD event, and the first to address HRV. Our results have shown that a short-term 14-day aquatic exercise training program is associated with specific HRV changes, which suggests that this mode of exercise is safe and may be beneficial in patients with CAD.

## Figures and Tables

**Table 1:** Baseline characteristics and between-group differences.

	Overall (*n* = 89)	Land-Based Group (*n* = 30)	Water-Based Group (*n* = 29)	Control Group (*n* = 30)	*p*
Age, years; mean (SD)	59.9 (8.2)	62.4 (7.6)	56.7 (8.4)	60.6 (8.3)	0.026
Gender (women, %)	20 (25%)	9 (30%)	5 (17.2%)	6 (20%)	0.464
CAD type					
MI + PCI	60 (67.4%)	19 (63.3%)	17 (58.6%)	24 (80%)	0.523
PCI	3 (3.4%)	1 (3.3%)	2 (6.9%)	0 (0%)	
CABG	9 (10.1%)	3 (10%)	3 (10.3%)	3 (10%)	
MI + CABG	17 (19.1%)	7 (23.3%)	7 (24.1%)	3 (19%)	
Risk factors					
Arterial hypertension	52 (58.4%)	18 (60%)	14 (48.3%)	20 (66.7%)	0.350
Dyslipidemia	65 (73%)	19 (63.3%)	22 (75.9%)	24 (80%)	0.318
Family history	53 (59.6%)	19 (63.3%)	18 (62.1%)	16 (53.3%)	0.692
Obesity	20 (22.5%)	6 (20%)	7 (24.1%)	7 (23.3%)	0.921
Diabetes mellitus	15 (16.9%)	3 (10%)	5 (17.2%)	7 (23.3%)	0.385
Physical inactivity	44 (49.4%)	17 (56.7%)	15 (51.7%)	12 (40%)	0.415
Smoking	48 (53.9%)	15 (59%)	16 (55.2%)	17 (53.9%)	0.863
Therapy					
Aspirin	86 (96.6%)	29 (96.7%)	29 (100%)	28 (93.3%)	0.366
Beta blockers	76 (85.4%)	26 (86.7%)	24 (82.8%)	26 (86.7%)	0.887
P2Y12 inhibitors	45 (50.6%)	19 (63.3%)	17 (58.6%)	9 (39%)	0.020
Statins	79 (88.8%)	26 (86.7%)	26 (89.7%)	27 (90%)	0.906
ACE/ARB	72 (80.9%)	%	22 (75.9%)	26 (86.7%)	0.566

SD—standard deviation; CAD—coronary artery disease; MI—myocardial infarction; PCI—percutaneous coronary intervention; CABG—coronary artery by-pass graft; ACE—angiotensin-converting-enzyme inhibitor; ARB—angiotensin II receptor blockers.

**Table 2:** HRV parameters (time domain) before and after the intervention period.

	Land-Based Exercise Group			Water-Based Exercise Group			Control Group			*p* *
	Baseline	After	*p*	Baseline	After	*p*	Baseline	After	*p*	
SDNN	37,1 (15.6)	35 (16)	0.504	39 (16.6)	35.7 (18.3)	0.364	35.4 (13.4)	39.3 (12.3)	0.120	0.256
aSDANN	23.8 (19.2–31.7)	21.0 (17.0–32.5)	0.338	26.5 (19.7–31.7)	23.4 (17.4–27.5)	0.354	25.2 (18.9–34)	26 (22.7–35.9)	0.274	0.306
NNx	83.7 (142.9)	49.9 (97)	0.175	41.6 (59.4)	53.9 (110.9)	0.515	65.2 (144.5)	113.7 (184.2)	0.080	0.052
pNNx	0.4 (0–8.2)	0.8 (0.2–2.5)	0.029	0.8 (0.5–5.6)	0.3 (0.1–2.7)	0.324	1.2 (0.2–6.4)	1.1 (0.3–17.9)	0.276	0.080
RMSSD	15.2 (10.5–30.7)	18.0 (10–24)	0.126	18.9 (14.7–25.1)	14.4 (11.8–19.7)	0.182	17.6 (14.4–26)	19.3 (14.8–37.5)	0.307	0.095
SDNNi	197 (165–227)	186 (172–227)	0.545	200 (170–220)	170 (150–209)	0.166	195 (21.9–226)	213 (163–240)	0.212	0.124
meanHR	62.6 (9.5)	63.5 (8.8)	0.422	64.1 (8.7)	68.3 (9.9)	0.012	57.3 (7.5)	56.5 (7.4)	0.518	0.002
sdHR	2.3 (0.7)	2.3 (0.9)	0.925	2.6 (1)	2.7 (0.9)	0.885	1.9 (0.7)	2.1 (0.6)	0.121	0.524
HRVti	10.2 (1,9)	9.6 (2.2)	0.278	10.9 (1.8)	10.2 (1.7)	0.137	10.1 (2.3)	10.4 (1.8)	0.595	0.380
TINN	148.9 (64.6)	140.7 (66.7)	0.583	156.5 (64.9)	136.8 (67.5)	0.212	141.2 (66.8)	156.5 (59.7)	0.224	0.248

Data are shown as mean (standard deviation) or median (Q1–Q3). IBI—inter-beat interval; SDNN—standard deviation of all normal R–R (NN) intervals; SDANN—standard deviation of the average NN interval calculated over a short period; NNx—number of valid adjacent NN values not separated by data breaks; pNNx—proportion of valid adjacent RR values not separated by data breaks; rMSSD—root mean square of successive NN interval differences; SDNNi—mean of the standard deviations of all the NN intervals for each 5 min segment; SDHR—standard deviation of heart rate; HRVti—heart rate variability triangular index; TINN—triangular interpolation interval histogram; *p* *—*p* between groups (ANCOVA).

**Table 3:** HRV parameters (frequency domain: Welch) before and after the intervention period.

	Land-Based Exercise Group			Water-Based Exercise Group			Control Group			*p* *
	Baseline	After	*p*	Baseline	After	*p*	Baseline	After	*p*	
TP (ms^2^)	828.2 (506.2–1431.9)	707.5 (430.4–1545)	0.527	900 (674–1543.8)	786.9 (445.5–1013.3)	0.228	872.2 (538–1788.4)	1121.6 (916–2036.4)	0.107	0.090
nLF	0.62 (0.55–0.76)	0.67 (0.44–0.73)	0.427	0.72 (0.54–0.8)	0.75 (0.68–0.85)	0.062	0.63 (0.46–0.75)	0.61 (0.44–0.74)	0.857	0.163
nHF	0.38 (0.24–0.45)	0.34 (0.27–0.56)	0.427	0.28 (0.2–0.46)	0.25 (0.15–0.32)	0.062	0.37 (0.25–0.54)	0.39 (0.26–0.56)	0.857	0.163
LF/HF	1.6 (1.2–3.2)	2 (0.8–2.7)	0.510	2.6 (1.2–4)	3 (2.1–5.5)	0.036	1.7 (0.8–3)	1.5 (0.8–2.8)	0.981	0.066
peakVLF (Hz)	0 (0–0)	0 (0–0)	0.317	0 (0–0.01)	0 (0–0)	0.046	0 (0–0)	0 (0–0)	0.317	0.065
peakLF (Hz)	0.07 (0.04–0.12)	0.09 (0.04–0.13)	0.715	0.05 (0.04–0.1)	0.07 (0.04–0.1)	0.970	0.07 (0.05–0.11)	0.09 (0.04–0.12)	0.777	0.949
peakHF (Hz)	0.26 (0.24–0.3)	027 (0.23–0.3)	0.914	0.25 (0.18–0.31)	0.27 (0.19–0.31)	0.909	0.24 (0.2–0.3)	0.24 (0.2–0.29)	0.548	0.873

Data are shown as mean (standard deviation) or median (Q1–Q3). TP—total power; LF—power in the low-frequency range; HF—power in the high-frequency range; LF/HF—ratio of low- and high-frequency power; peakVLF—peak frequency of the very-low-frequency band; peakLF—peak frequency of the low-frequency band; peakHF—peak frequency of the high-frequency band. *p* *—*p* between groups (ANCOVA).

**Table 4:** HRV parameters (Poincaré and non-linear) before and after the intervention period.

	Land-Based Exercise Group			Water-Based Exercise Group			Control Group			*p* *
	Baseline	After	*p*	Baseline	After	*p*	Baseline	After	*p*	
SD1	10.8 (7.4–21.7)	12.7 (7.1–17)	0.130	13.3 (10.4–17.8)	10.2 (8.4–13.9)	0.187	12.4 (10.2–18.4)	13.6 (10.5–26.6)	0.307	0.930
SD2	44.4 (34.7–56.6)	40.9 (33.9–56)	0.642	46.8 (37–73.8)	42.6 (31.7–51)	0.372	42.5 (32.7–60.6)	48.9 (42.9–62.1)	0.138	0.383
SampEn	2.1 (1.7–2.2)	1.9 (1.7–2.1)	0.776	2 (1.7–2.2)	1.8 (1.6–2)	0.096	2.1 (1.7–2.5)	2.1 (1.9–2.4)	0.789	0.900
DFA *α1*	1.1 (0.9–1.4)	1.1 (0.9–1.3)	0.545	1.2 (1.1–1.4)	1.3 (1.2–1.5)	0.043	1.1 (0.8–1.3)	1.1 (0.9–1.3)	0.764	0.205

Data are shown as mean (standard deviation) or median (Q1–Q3). SD1—Poincaré plot standard deviation perpendicular to the line of identity; SD2—Poincaré plot standard deviation along the line of identity; SampEn—sample entropy; DFA α1—short-term scaling exponent obtained by detrended fluctuation analysis; *p* *—*p* between groups (ANCOVA). Main changes of time-domain, frequency-domain, and non-linear HRV parameters are depicted as Supplementary Figure S1, Figure S2, and Figure S3, respectively.

## Data Availability

Data available upon request.

## Supplementary Materials

The following supporting information can be downloaded at: https://www.mdpi.com/article/10.3390/jcdd9080251/s1, Table S1: Complete set of measurements of HRV parameters (IBI and time-domain) for the three study groups at baseline and after training; Table S2: Complete set of measurements of HRV parameters (frequency domain: Welch) for the three study groups at baseline and after training; Table S3: Complete set of measurements of HRV parameters (Poincaré and nonlinear) for the three study groups at baseline and after training; Figure S1: Change in time-domain HRV parameters for the each study groups at baseline and after training; Figure S2: Change in frequency domain: Welch–HRV parameters for the three study groups at baseline and after training; Figure S3: Change in Poincaré and nonlinear HRV parameters for the three study groups at baseline and after training
