# Supplementary material for: The Effect of Aquatic Exercise Training on Heart Rate Variability in Patients with Coronary Artery Disease
Source: J Cardiovasc Dev Dis. 2022 Aug 6;9(8):251. doi: 10.3390/jcdd9080251 (PMC9409327; doi:10.3390/jcdd9080251)
Supplement: Supplementary file 1 [file jcdd-09-00251-s001.zip › jcdd-1831031-supplementary.pdf]

Table S1: Complete set of measurements of HRV parameters (IBI and time-domain) for the three study groups at baseline and after training.

Variables	Land-exercise group (I)				Water-exercise group (II)				Control group (III)				p*
	Before	After	Δ	p	Before	After	Δ	p	Before	After	Δ	p	
**IBI** (count)	1388 (1196 - 1487)	1423 (1198 - 1553)	-35,0	0.989	1317.5 (1174 - 1557)	1426 (1261 - 1700)	-108,5	0.036	1237 (1092 - 1425)	1156 (1123 - 1385)	81,0	0.186	0,.05
**Outliers** (count)	14 (9 - 20)	12 (9 - 19)	2,0	0.891	12.5 (9 - 20)	13 (7 - 18)	-0,5	0.322	10 (6 - 18)	11 (7 - 20)	-1,0	0.411	0.475
**Max** (ms)	1088.2 (175.5)	1085.4 (171.6)	3,2	0.883	1068.8 (153.6)	1007 (158.5)	61,8	0.025	1173,2 (164.6)	1194.8 (150.1)	-21,6	0.389	0.004 (II-III))
**Min** (ms)	870 (794 - 908)	828 (746 - 906)	42,0	0.619	808 (754 - 884)	768.6 (672 - 870)	39,4	0.019	938 (836 - 1040)	946 (848 - 1056)	-8,0	0.919	0.116
**Mean** (ms)	979.9 (142,4)	963.9 (137)	16,.	0.341	959.0 (132,2)	898.3 (132,7)	60,7	0.014	1065,1 (136.7)	1079.8 (133,6)	-14,7	0.473	0.002 (II-III)
**Median** (ms)	981 (142.7)	964.5 (137.5)	16,5	0.328	956.4 (133.6)	900.7 (135.5)	55,7	0.015	1065.7 (135.8)	1081.9 (133.3)	-16,2	0.425	0.002 (II-III)
**SDNN** (ms)	37,1 (15.6)	35 (16)	2,1	0.504	39 (16.6)	35,7 (18.3)	3,3	0.364	35.4 (13.4)	39.3 (12.3)	-3,9	0.120	0.256
**SDANN** (ms)	23.8 (19.2 – 31.7)	21 (17 – 32.5)	2,8	0.338	26.5 (19.7 – 31.7)	23.4 (17.4 – 27.5)	3,1	0.354	25.2 (18.9 - 34)	26 (22.7 – 35.9)	-0,8	0.274	0.306
**NNx** (count)	83.7 (142.9)	49.9 (97)	33,8	0.175	41.6 (59.4)	53.9 (110.9)	-12,3	0.515	65.2 (144.5)	113.7 (184.2)	-49,5	0.080	0.052
**pNNx** (%)	0.4 (0 – 8.2)	0.8 (0.2 – 2.5)	-0,4	0.029	0.8 (0.5 – 5.6)	0.3 (0.1 – 2.7)	0,5	0.324	1.2 (0.2 – 6.4)	1.1 (0.3 – 17.9)	0,1	0.276	0.080
**RMSSD** (ms)	15,2 (10.5 – 30.7)	18 (10 - 24)	-2,8	0.126	18.9 (14.7 – 25.1)	14.4 (11.8 – 19.7)	4,5	0.182	17.6 (14.4 - 26)	19.3 (14.8 – 37.5)	-1,7	0.307	0.095
**SDNNi** (ms)	196.9 (164.8 – 226.8)	185.8 (171.9 – 226.6)	11,1	0.545	199.7 (169.6 – 219.6)	170.4 (150.5 – 209.6)	29,3	0.166	195 (21.9 - 226)	213.6 (163.6 – 240.1)	-18,6	0.212	0.124
**meanHR** (bpm)	62.6 (9.5)	63.5 (8.8)	-0,9	0.422	64.1 (8.7)	68.3 (9.9)	-4,2	0.012	57.3 (7.5)	56.5 (7.4)	0,8	0.518	0.002 (II-III)
**sdHR** (bpm)	2.3 (0.7)	2.3 (0.9)	0,0	0.925	2.6 (1)	2.7 (0.9)	-0,1	0.885	1.9 (0.7)	2.1 (0.6)	-0,2	0.121	0.524
**HRVti** (ms)	10.2 (1,9)	9.6 (2.2)	0,6	0.278	10.9 (1.8)	10.2 (1.7)	0,7	0.137	10.1 (2.3)	10.4 (1.8)	-0,3	0.595	0.380
**TINN** (ms)	148.9 (64.6)	140.7 (66.7)	8,2	0.583	156.5 (64.9)	136.8 (67.5)	19,7	0.212	141.2 (66.8)	156.5 (59.7)	-15,3	0.224	0.248

Data are shown as mean (standard deviation) or median (Q1–Q3). *IBI* – inter-beat interval; *SDNN* – standard deviation of all normal R–R (NN) intervals; *SDANN* – standard deviation of the average NN interval calculated over a short period; *NNx* – number of valid adjacent NN values not separated by data breaks; *pNNx* – proportion of valid adjacent RR values not separated by data breaks; *rMSSD* – root mean square of successive NN interval differences; *SDNNi* – mean of the standard deviations of all the NN intervals for each 5 min segment; *SDHR* – standard deviation of heart rate; *HRVti* – heart rate variability triangular index; *TINN* – triangular interpolation interval histogram; *p** – *p* between groups (ANCOVA).

Table S2: Complete set of measurements of HRV parameters (frequency domain: Welch) for the three study groups at baseline and after training.

Variables	Land-exercise group (I)				Water-exercise group (II)				Control group (III)				P*
	Before	After	△	p	Before	After	△	p	Before	After	△	p	
**aVLF** (ms^2)	477 (324.8 – 691.7)	488.3 (298.5 – 832.1)	-11,3	0.946	560.6 (367 – 9239)	506.2 (285.5 – 747.8)	54,4	0.439	532.3 (349.8 – 1074.7)	699 (463 – 1176.5)	-166,7	0.068	0,171
**aLF** (ms^2)	182.8 (87.6 – 375.4)	114.2 (63 – 320.4)	68,6	0.253	229.5 (114.6 – 442.7)	180 (87.5 - 345)	49,5	0.304	170.9 (104.9 – 380.6)	217.5 (137.5 – 394.1)	-46,6	0.259	0,246
**aHF** (ms^2)	79.2 (41 – 244.6)	88.9 (35.3 – 160.9)	-9,7	0.025	85 (64.4 – 175.6)	58.7 (32.4 – 114.5)	26,3	0.052	104.3 (44.1 – 272.3)	128.3 (60.7 – 430.8)	-24	0.290	0,033 (I-III, II-III)
**aTotal** (ms^2)	828.8 (506.2 – 1431.9)	707,5 (430,4 – 1545)	121,3	0.527	900 (674 – 1543.8)	786.9 (445.5 – 1013.3)	113,1	0.228	872.2 (538.3 – 1788.4)	1121.6 (916 – 2036.4)	-249,4	0.107	0,090
**pVLF** (%)	67.5 (50.7 – 71.9)	70.5 (66.2 – 75.6)	-3	0.020	61,9 (55.6 – 71.8)	68.1 (58.1 – 73.6)	-6,2	0.443	60.4 (51.9 – 75.3)	63.7 (56.2 – 74.6)	-3,3	0.524	0,466
**pLF** (%)	22.7 (15.7 – 28.9)	18.5 (11.9 – 24.9)	4,2	0.038	24.7 (14.2 – 32.9)	22.8 (16.7 – 32.5)	1,9	0.620	20 (15.7 – 26.1)	20.1 (15 – 24.1)	-0,1	0.665	0,322
**pHF** (%)	9.8 (6.7 – 18.8)	8.7 (7.1 – 17.6)	1,1	0.300	9.8 (6.1 - 15)	7.3 (5 – 11.4)	2,5	0.089	15.9 (5.7 – 26.5)	13.3 (8.5 – 19.6)	2,6	0.692	0,897
**nLF** (%)	0.62 (0.55 – 0.76)	0.67 (0.44 – 0.73)	-0,05	0.427	0.72 (0,54 – 0.8)	0.75 (0.68 – 0.85)	-0,03	0.062	0.63 (0.46 – 0.75)	0.61 (0.44 – 0.74)	0,02	0.857	0,163
**nHF** (%)	0.38 (0.24 – 0.45)	0.34 (0.27 – 0.56)	0,04	0.427	0.28 (0.2 – 0.46)	0.25 (0.15 – 0.32)	0,03	0.062	0.37 (0,25 – 0.54)	0.39 (0.26 – 0.56)	-0,02	0.857	0,163
**LF / HF**	1.6 (1.2 – 3.2)	2 (0.8 – 2.7)	-0,4	0.510	2.6 (1.2 - 4)	3 (2.1 – 5.5)	-0,4	0.036	1.7 (0.8 - 3)	1.5 (0.8 – 2.8)	0,2	0.981	0,066
**peakVLF** (Hz)	0 (0 - 0)	0 (0 - 0)	0	0.317	0 (0 – 0.01)	0 (0 - 0)	0	0.046	0 (0 - 0)	0 (0 - 0)	0	0.317	0,065
**peakLF** (Hz)	0.07 (0.04 – 0.12)	0.09 (004 – 0.13)	-0,02	0.715	0.05 (0.04 – 0.1)	0.07 (0.04 – 0.1)	-0,02	0.970	0.07 (0.05 – 0.11)	0.09 (0.04 – 0.12)	-0,02	0.777	0,949
**peakHF** (Hz)	0.26 (0.24 – 0.3)	0.27 (0.23 – 0.3)	-0,01	0.914	0,25 (0,18 - 0,31)	0.27 (0.19 – 0.31)	-0,02	0.909	0.24 (0.2 – 0.3)	0.24 (0.2 – 0.29)	0	0.548	0,873

Data are shown as mean (standard deviation) or median (Q1-Q3). aVLF – absolute value in very low frequency; aLF – absolute value in low frequency; aHF -absolute value in high frequency; pVLF – power of the very low frequency band; pLF – power of the low frequency band; pHF – power of the high frequency band; aTotal – total power, variance of all NN intervals; nLF – power in the low-frequency range, normalized; nHF – power in the high-frequency range, normalized; LF/HF -ratio of normalized low-and high-frequency power; peakVLF – peak frequency of the very low-frequency band; peakLF – peak frequency of the low-frequency band; peakHF – peak frequency of the high-frequency band. p – p between groups (ANCOVA).*

Table S3: Complete set of measurements of HRV parameters (Poincaré and nonlinear) for the three study groups at baseline and after training.

Variables	Land-exercise group (I)				Water-exercise group (II)				Control group (III)				P*
	Before	After	△	p	Before	After	△	p	Before	After	△	p	
**SD1** (ms)	10.8 (7.4 – 21.7)	12.7 (7.1 - 17)	-1,9	0.130	13.3 (10.4 – 17.8)	10.2 (8.4 – 13.9)	3,1	0.187	12.4 (10.2 – 18.4)	13.6 (10.5 – 26.6)	1,9	0.307	0,093
**SD2** (ms)	44.4 (34.7 – 56.6)	40.9 (33.9 - 56)	3,5	0.642	46.8 (37 – 73.8)	42.6 (31.7 - 51)	4,2	0.372	42.5 (32.7 – 60.6)	48.9 (42.9 – 62.1)	-6,4	0.138	0,383
**SampEn**	2.1 (1.7 – 2.2)	1.9 (1.7 – 2.1)	0,2	0.776	2 (1.7 – 2.2)	1.8 (1.6 - 2)	0,2	0.096	2.1 (1.7 – 2.5)	2.1 (1.9 – 2.4)	0	0.789	0,090
**Alpha**	1 (0.9 – 1.1)	1.1 (1.1 – 1.1)	-0,1	0.017	1 (1 – 1.1)	1.1 (1 – 1.1)	-0,1	0.525	1 (1 – 1.1)	1 (1 – 1.1)	0	0.962	0,104
**Alpha1**	1.1 (0.9 – 1.4)	1.1 (0.9 – 1.3)	0	0.545	1.2 (1.1 – 1.4)	1.3 (1.2- 1.5)	-0,1	0.043	1.1 (0.8 – 1.3)	1.1 (0.9 – 1.3)	0	0.764	0,205
**Alpha2**	1 (0.9 - 1)	1.1 (0.9 – 1.1)	-0,1	0.003	1 (0.9 – 1.1)	1 (0.9 - 1)	0	0.970	1 (0.9 – 1.1)	1 (0.9 - 1)	0	0.764	0,029 (I-II; I-III)

Data are shown as mean (standard deviation) or median (Q1-Q3). *SD1* – poincare plot standard deviation perpendcularthe line of identity; *SD2* – Poincare plot standard deviation along the line of identity; *SampEn* – sample entropy; 〈*1* – detrended fluctuation analysis which describes short – time fluctuation; 〈*2* – detrended fluctuation analysis, which describes long-term fluctuations.

*p** – *p* between groups (ANCOVA).

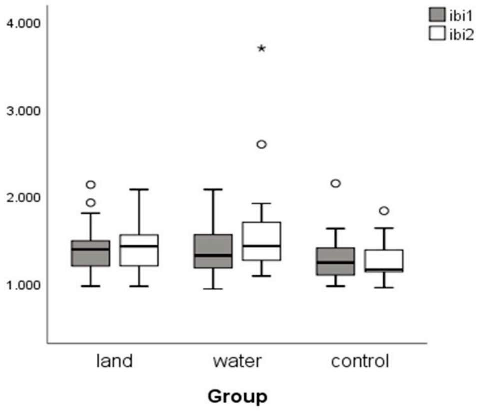

ibi1
ibi2
4.000
3.000
2.000
1.000
land
water
control
Group

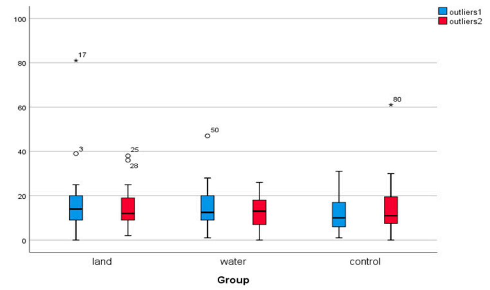

outliers1
outliers2
land
water
control
Group

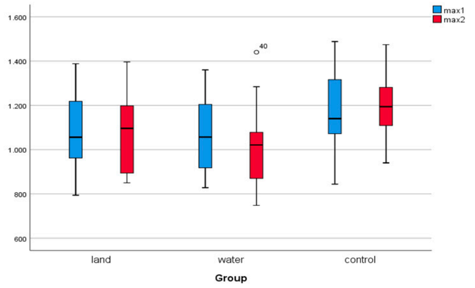

max1
max2
land
water
control
Group

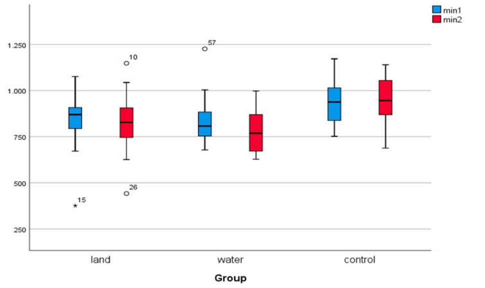

min1
min2
land
water
control
Group

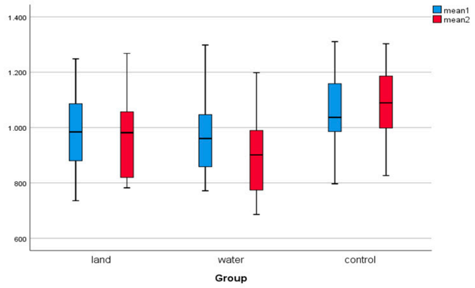

mean1
mean2
land
water
control
Group

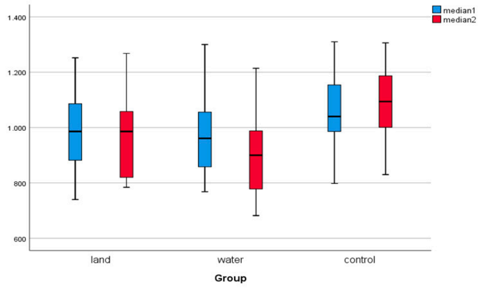

median1
median2
land
water
control
Group

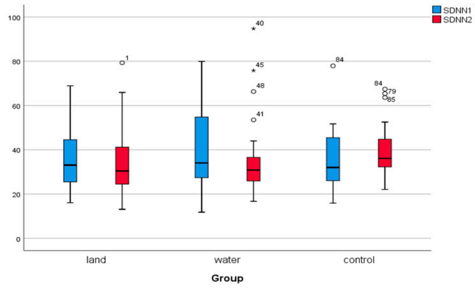

SDNN1
SDNN2
land
water
control
Group

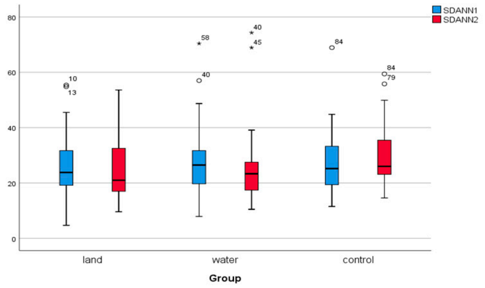

SDANN1
SDANN2
land
water
control
Group

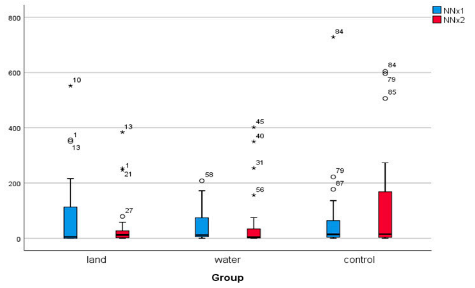

NNx1
NNx2
land
water
control
Group

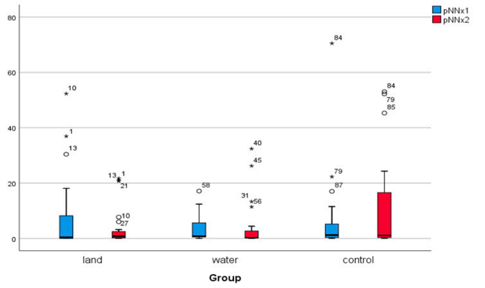

pNNx1
pNNx2
land
water
control
Group

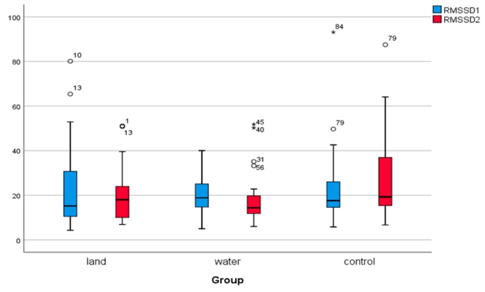

RMSSD1
RMSSD2
land
water
control
Group

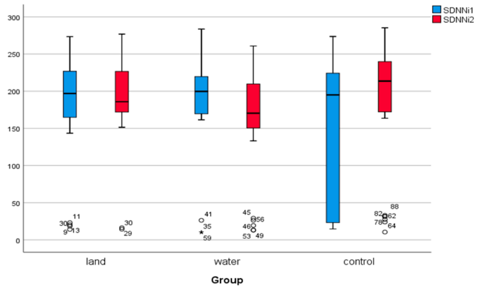

SDNNi1
SDNNi2
land
water
control
Group

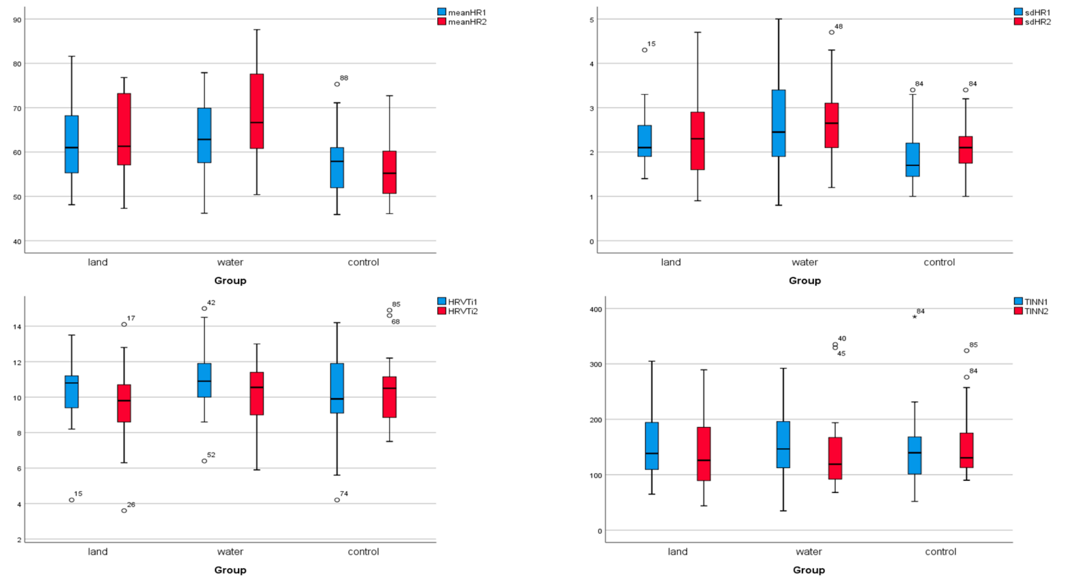

Figure S1. Change in time-domain HRV parameters for the each study groups at baseline and after training.

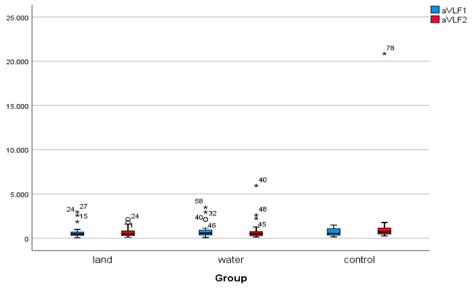
aVLF1
aVLF2
25.000
20.000
15.000
10.000
5.000
0
land
water
control
Group
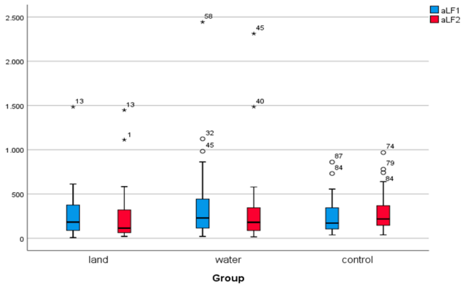
aLF1
aLF2
2.500
2.000
1.500
1.000
500
0
land
water
control
Group
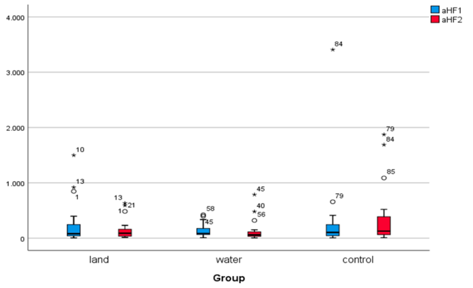
aHF1
aHF2
4.000
3.000
2.000
1.000
0
land
water
control
Group
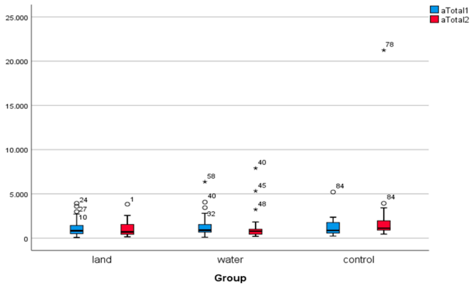
aTotal1
aTotal2
25.000
20.000
15.000
10.000
5.000
0
land
water
control
Group
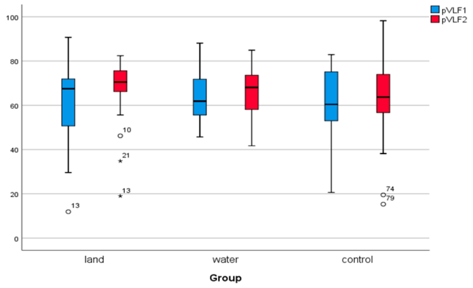
pVLF1
pVLF2
100
80
60
40
20
0
land
water
control
Group
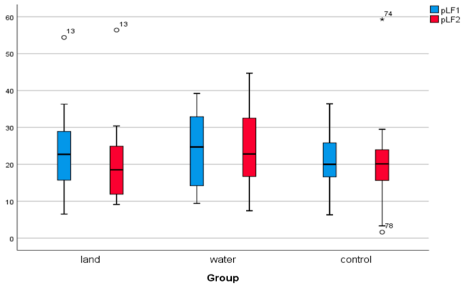
pLF1
pLF2
60
50
40
30
20
10
0
land
water
control
Group
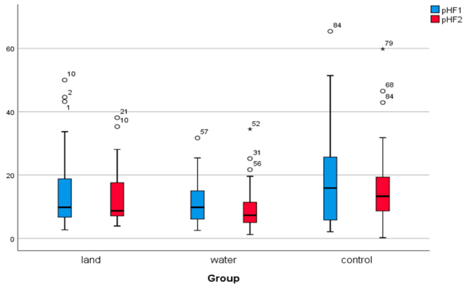
pHF1
pHF2
60
40
20
0
land
water
control
Group
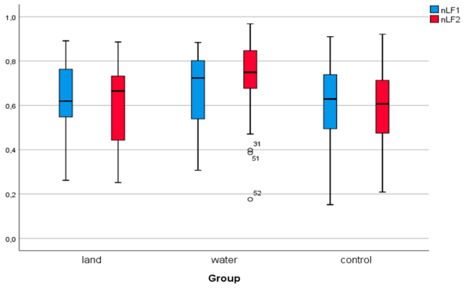
nLF1
nLF2
1,0
0,8
0,6
0,4
0,2
0,0
land
water
control
Group
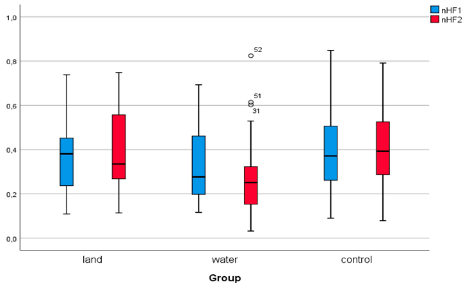
nHF1
nHF2
1,0
0,8
0,6
0,4
0,2
0,0
land
water
control
Group
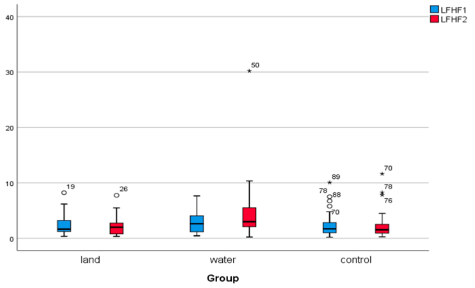
LFHF1
LFHF2
40
30
20
10
0
land
water
control
Group
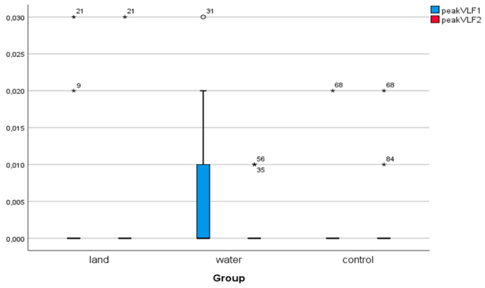
peakVLF1
peakVLF2
0,030
0,025
0,020
0,015
0,010
0,005
0,000
land
water
control
Group
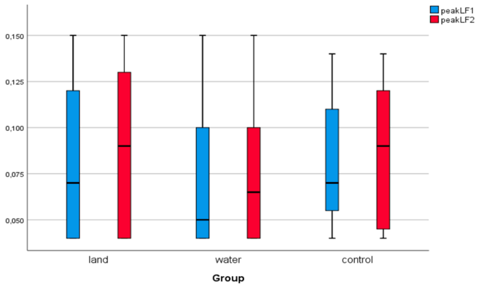
peakLF1
peakLF2
0,150
0,125
0,100
0,075
0,050
land
water
control
Group

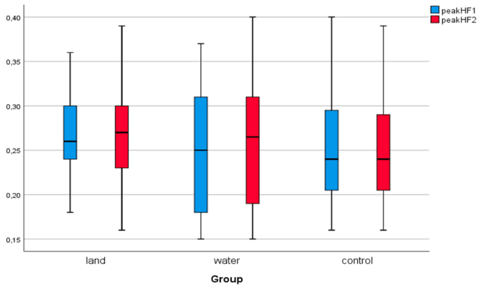

Figure S2. Change in frequency domain: Welch – HRV parameters for the three study groups at baseline and after training.

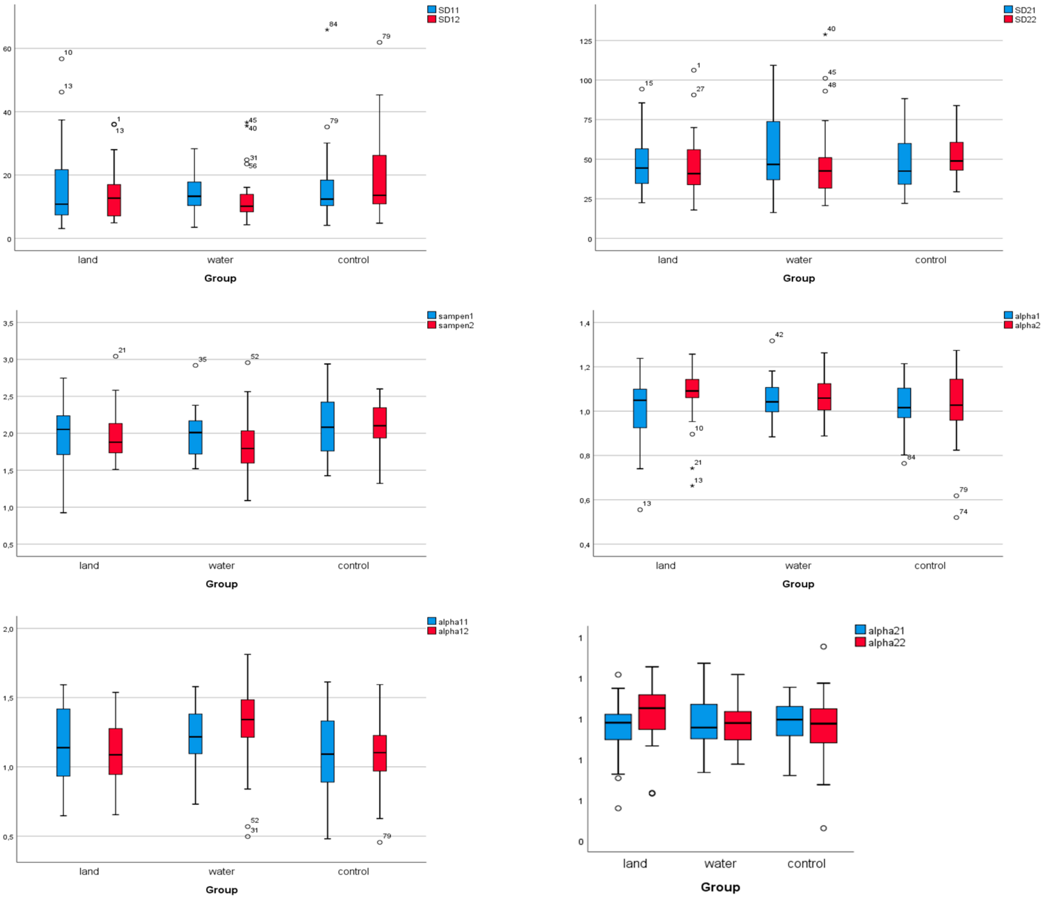

Figure S3. Change in Poincaré and nonlinear HRV parameters for the three study groups at baseline and after training.
